# Framework for creating a qualified medical device development tool of autoinjectors

**DOI:** 10.3389/fmedt.2023.1281403

**Published:** 2023-11-24

**Authors:** Marlon Luca Machal

**Affiliations:** Faculty of Medicine and Health Technology, Tampere University, Tampere, Finland

**Keywords:** autoinjectors, medical device development tool, primary functions, framework, remote treatments

## Abstract

**Objectives:**

Autoinjectors are pivotal for precise self-administration of medications across a wide range of medical conditions. Nevertheless, the absence of a dedicated Medical Device Development Tool (MDDT) for autoinjectors represents a gap that may result in variations in the quality and regulatory compliance of autoinjectors as components of combination products. This research aim is to utilize the recently introduced Primary Functions outlined in ISO 11608-1:2022 with the title “Needle-based injection systems for medical use. Requirements and test methods. Part 1: Needle-based injection systems” to create a comprehensive MDDT framework tailored specifically for autoinjectors.

**Methods:**

To support the creation of the framework, the analysis of the FDA MDDTs that were already approved, FDA's design controls regulations, FDA's guidance related to autoinjectors, and the Primary functions outlined in ISO 11608-1:2022 were utilized.

**Results:**

The research identifies the Primary Functions in autoinjector to be Holding Force, Cap Removal Force, Activation Force, Extended Needle Length, Injection Time, Dose Accuracy and Needle Guard Lockout. Leveraging these Primary Functions and the FDA's MDDT approach, the research aims to bridge the gap by proposing a structured framework for the development of a specific MDDT tailored to autoinjectors.

**Conclusion:**

This study presents a MDDT framework tailored to the development of autoinjectors for drug delivery. This framework provides a structured methodology to support predictability and effectiveness of the autoinjector development and support regulatory review process, thereby expediting FDA approval for autoinjectors as part of combination product.

## Introduction

Autoinjectors are defined as devices designed to administer a pre-measured dose of medication through self-injection usually subcutaneously or intramuscularly (1). Autoinjectors gained and continue to gain prominence due to their convenience, precision, and ease of use. These devices cater to a diverse range of medical conditions, from chronic diseases to emergency treatments, underscoring their significance in patient care (2–8). By enabling remote or self-administration of treatments, autoinjectors confer a distinct advantage, especially in situations resembling the Covid-19 pandemic, where patient hospital visits are restricted or minimized. Forecasts indicate a substantial increase of the autoinjectors market, projecting a surge from 2.88 billion to 12.67 billion by the year 2030 (9). Autoinjectors development has experienced significant progress, leading to the emergence of innovative solutions that enhance patient care and treatment efficacy (10–12). The FDA's Medical Device Development Tool (MDDT) has emerged as a pivotal resource that revolutionizes the development and evaluation of various medical devices (13). The FDA's MDDT serves as an indispensable basis for the development of various medical devices. MDDT is a method, material, or measurement used to develop and evaluate the safety, effectiveness and performance of a medical device (13). This tool needs to be scientifically substantiated and may be qualified for use in device evaluation and support regulatory decision-making (13). By offering a standardized approach, the MDDT ensures consistency in development practices, resulting in improved device quality and patient safety. Autoinjectors, as integral components of combination products, necessitate dedicated attention because of their crucial in delivering medications safely and effectively. Yet, there is no MDDT that is used for autoinjectors. The lack of a dedicated MDDT for autoinjector can result in variability in combination product quality, safety, and regulatory compliance. This research employed the newly introduced Primary Functions outlined in ISO 11608-1:2022 (14) to develop a framework for creating a proficient MDDT tool. This Framework can serve as guidance for autoinjector manufacturers regarding the MDDT requirements for autoinjectors.

## Material and methods

The material of this research is the pre-existing MDDTs that have been approved by the FDA (13), in conjunction with the Primary Functions of autoinjectors (14), the FDA's design controls regulations and the FDA's guidance related to autoinjectors. ISO 11608-1:2022 defined Primary Functions as those functions that are essential for ensuring the autoinjector's safety and effectiveness (14). Notably, no research has investigated the recently introduced Primary Functions outlined in ISO 11608-1:2022 concerning the MDDT for autoinjectors. By assessing the Primary Functions of autoinjectors, a structured framework can be created to establish a qualified MDDT tailored specifically for autoinjectors.

## Results

A mere 14 MDDTs have received FDA approval (13). These approved MDDTs have been found suitable for deployment across range of the FDA's defined Product Areas, encompassing Biostability, Orthopedic, MR Safety Labeling, Toxicology, Biocompatibility, Plastic Surgery, Dermatology, Imaging, Ophthalmology, Active implanted medical devices, Cybersecurity, Automated Insulin Dosing, Neurology, and Cardiology (13). Yet, autoinjectors were not included in the list of approved product areas (13).

The Primary Functions of autoinjectors were concluded from recent literature review studies focusing on autoinjectors (15). These identified Primary Functions, crucial for guaranteeing the safety and effectiveness of autoinjectors, entail Holding Force, Cap Removal Force, Activation Force, Extended Needle Length, Injection Time, Dose Accuracy and Needle Guard Lockout.

## Discussion

As of May 30, 2022, the FDA recognized ISO 11608-1:2022 (16) and encouraged autoinjector manufacturers to adopt these ISO standards to demonstrate conformity with FDA requirements and testing methods pertaining to Needle-based injection systems for medical devices such as autoinjectors. These requirements and testing methods are based on the recently introduced Primary Functions outlined in ISO 11608-1:2022. Hence, these testing methods must ensure the accurate functioning of Holding Force, Cap Removal Force, Activation Force, Extended Needle Length, Injection Time, Dose Accuracy, and Needle Guard Lockout. The Primary Functions that have been identified in this research are open to discussion and require consensus between the FDA and the drug applicants in order to validate the proposed Primary Functions.

Considering the objectives of the MDDT which aims to streamline the processes encompassing design, manufacturing, testing, transporting, storing and regulatory approval for medical devices, manufacturers must focus on the critical components that ensures proper function of the autoinjectors. In cases where these components will not be designed or manufactured correctly, the Primary Functions of the autoinjector can be compromised, potentially resulting in the device's failure to administer medication effectively. Consequently, this failure can pose risks to both patients and healthcare professionals utilizing the autoinjector. Following the design control process (17–19) outlined in the FDA's medical device regulation section 820.30 (20) and integrating each Primary Functions of the autoinjector, manufacturers will be able to create a Design History File (DHF) that is compliant with the FDA requirements (20–22).

Autoinjectors documentations are part of the Common Technical Documentation (CTD) (23) that is required to be submitted to support the drug application filling to the FDA. Autoinjectors part belongs to the container closure system of the CTD (23). Pharmaceutical companies introducing new drugs via autoinjectors that is produced using MDDT are spared the need to commence efforts from scratch to establish a new DHF that is used for container closure system in CTD. The framework in Table 1 can serve as a guiding framework for autoinjector manufacturers, aiding them in developing a qualified MDDT by aligning Primary Functions and critical components of autoinjector.

**Table 1:** Framework for creating MDDT by using primary functions and critical components of autoinjector.

Qualified MDDT Framework			
1. Prefilled drug syringe	2. Critical components	3. Primary functions as per ISO 11608-1:2022	4. Design, manufacturing, testing, transporting, storing
Type of syringe, drug viscosity, drug flow rate, drug volume	Follow design control process as per 21CFR 820.30	Holding force	Document evidence in DHF as per 21CFR 820.30
		Cap removal force	
		Activation force	
		Extended needle length	
		Injection time	
		Dose accuracy	
		Needle guard lockout	

Autoinjectors represent needle-based injection systems comprising prefilled syringes filled with drugs (24, 25). The critical components of the autoinjector are determined by the viscosity, flow rate and volume of the drug (26–28). The proposed framework involves four sequential steps. The process consists of four key steps. First step, manufacturers of the autoinjector identify the appropriate syringe for drug filling, taking into account factors like drug viscosity, flow rate, and volume, as these factors impact the autoinjector's Primary Functions. Second step, the critical components of the autoinjector need to be identified as they must adhere to the design control process specified in the FDA's regulations and guidance documents (21–29). Third step, Primary Functions outlined in ISO 11608-1:2022 are employed to validate the correct operation of the identified critical components. Lastly, the fourth step revolves around recording document evidence of various aspects of design, production, testing, transportation, and storage, which collectively comprise the Design History File (DHF) (20) for the autoinjector. The result of MDDT is the creation of a singular DHF document that can be utilized to support the section of container closure systems within the drug application's common technical documentation to the FDA (23). Consequently, pharmaceutical companies will only need to provide to the FDA DHF substantiation that the autoinjector is manufactured using an FDA qualified MDDT. Additionally, a design verification report must be submitted to demonstrate the critical components conform with the Primary Functions in Table 1 as per ISO 11608-1:2022 (14).

The categorization of autoinjectors for subcutaneous or intramuscular self-injection is rooted in the identified Primary Functions that dictate their proper function. As a result, the suggested Product Area (13) associated with autoinjectors can be characterized as a Non-clinical Assessment Model (13). This classification underscores the significance of evaluating these devices within a non-clinical context, focusing on their fundamental functions and performance parameters to ensure they meet the required standards for self-administration (14). This approach allows for a comprehensive assessment of autoinjectors' functionality, safety, and effectiveness, setting the stage for their successful use in real-world clinical applications.

## Conclusion

By identifying critical components of an autoinjector based on factors like viscosity, flow rate and volume of the drug, manufacturers can ensure the precise functioning of autoinjectors. In the lack of MDDT dedicated for autoinjectors, the guidance offered by the framework illustrated in Table 1 represents a valuable tool for creating a thorough MDDT customized for autoinjectors, ultimately seeking qualification from the FDA. The MDDT's as Non-clinical Assessment Model (13) value lies in its ability to eliminate redundancy for pharmaceutical companies introducing existing or new drugs through autoinjectors. Manufacturers can demonstrate compliance by submitting relevant design verification reports that are deemed acceptable by the FDA, showing their commitment to ensuring the proper functionality of autoinjectors for a range of drugs. This will have the dual effect of accelerating time-to-market for pharmaceutical companies and affording patients' seamless access to treatments that empower them to manage their health conditions with ease, circumventing the necessity of frequent hospital visits.

## Data Availability

The original contributions presented in the study are included in the article/supplementary material. Further inquiries can be directed to the corresponding author.
